# Association between chronic obstructive pulmonary disease and ventricular arrhythmia: a nationwide population-based cohort study

**DOI:** 10.1038/s41533-021-00221-3

**Published:** 2021-02-12

**Authors:** Chun-Chao Chen, Cheng-Hsin Lin, Wen-Rui Hao, Chun-Chih Chiu, Yu-Ann Fang, Ju-Chi Liu, Li-Chin Sung

**Affiliations:** 1 Division of Cardiology, Department of Internal Medicine, Shuang Ho Hospital, Taipei Medical University, New Taipei City, Taiwan; 2 Taipei Heart Institute, Taipei Medical University, Taipei, Taiwan; 3 Cardiovascular Research Center, Taipei Medical University Hospital, Taipei, Taiwan; 4 Division of Cardiovascular Surgery, Department of Surgery, Shuang Ho Hospital, Taipei Medical University, New Taipei City, Taiwan; 5 Division of Cardiovascular Surgery, Department of Surgery, School of Medicine, College of Medicine, Taipei Medical University, Taipei, Taiwan; 6 Graduate Institute of Clinical Medicine, College of Medicine, Taipei Medical University, Taipei, Taiwan; 7 Division of Cardiology, Department of Internal Medicine, School of Medicine, College of Medicine, Taipei Medical University, Taipei, Taiwan

**Keywords:** Chronic obstructive pulmonary disease, Epidemiology

## Abstract

The ventricular arrhythmia (VA)–chronic obstructive pulmonary disease (COPD) association and related risk factors remain unclear. Using 2001–2012 data from National Health Insurance Research Database, we retrospectively reviewed 71,838 patients diagnosed as having COPD and 71,838 age- and sex-matched controls. After adjustments for comorbidities, medication, urbanization level, and monthly income, patients with COPD had higher incidence rates of VA than did the controls (adjusted hazard ratio [aHR] [95% confidence interval (CI)]: 1.45 [1.25–1.68]). More hospitalization or emergency visits because of acute COPD exacerbation (aHRs [95% CIs] for first, second, and third visits: 1.28 [1.08–1.50], 1.75 [1.32–2.32], and 1.88 [1.46–2.41], respectively) and asthma–COPD overlap (aHR [95% CI]: 1.49 [1.25–1.79]) were associated with high VA risk in patients with COPD. In the multivariate analysis, heart failure (aHR [95% CI]: 2.37 [1.79–3.14]), diabetes (aHR [95% CI]:1.64 [1.29–2.08]), age ≥75 (aHR [95% CI]: 2.48 [1.68–3.67]), male (aHR [95% CI]: 1.69[1.34–2.12]), and class III antiarrhythmic drug use (aHR [95% CI]: 2.49 [1.88–3.28]) are the most significant risk factors of new onset of VA in patients with COPD.

## Introduction

Chronic obstructive pulmonary disease (COPD) is a severe lung disease and a major cause of mortality and morbidity worldwide^1^. The causes of death in patients with COPD include acute or chronic respiratory failure, infection, coronary artery disease (CAD), heart failure (HF), and cardiac arrhythmia^2,3^. The major risk factors for mortality in patients with COPD are acute myocardial infarction (AMI) and underlying CAD^2^. Because COPD is associated with systemic inflammation, which initiates or aggravates comorbid diseases, cardiovascular disease and arrhythmia risks have been reported to be associated with COPD^4–7^. In previous studies, supraventricular arrhythmia, particularly atrial fibrillation, was the most common cardiac arrhythmia in patients with COPD^6^. However, in patients with acute COPD exacerbation, the most common arrhythmia was reported to be ventricular premature beats^8^. In addition, COPD and its severity have been reported as independent risk factors for ventricular tachycardia^7,9^. However, because of COPD complexities, such as comorbidities, prescribed medications, and asthma–COPD overlap (ACO) risk, the COPD–ventricular arrhythmia (VA) association remains unclear. In this study, VA risk in patients with COPD was assessed by analyzing nationwide population-based data.

## Results

### Baseline characteristics of the study population

**Fig. 1:**
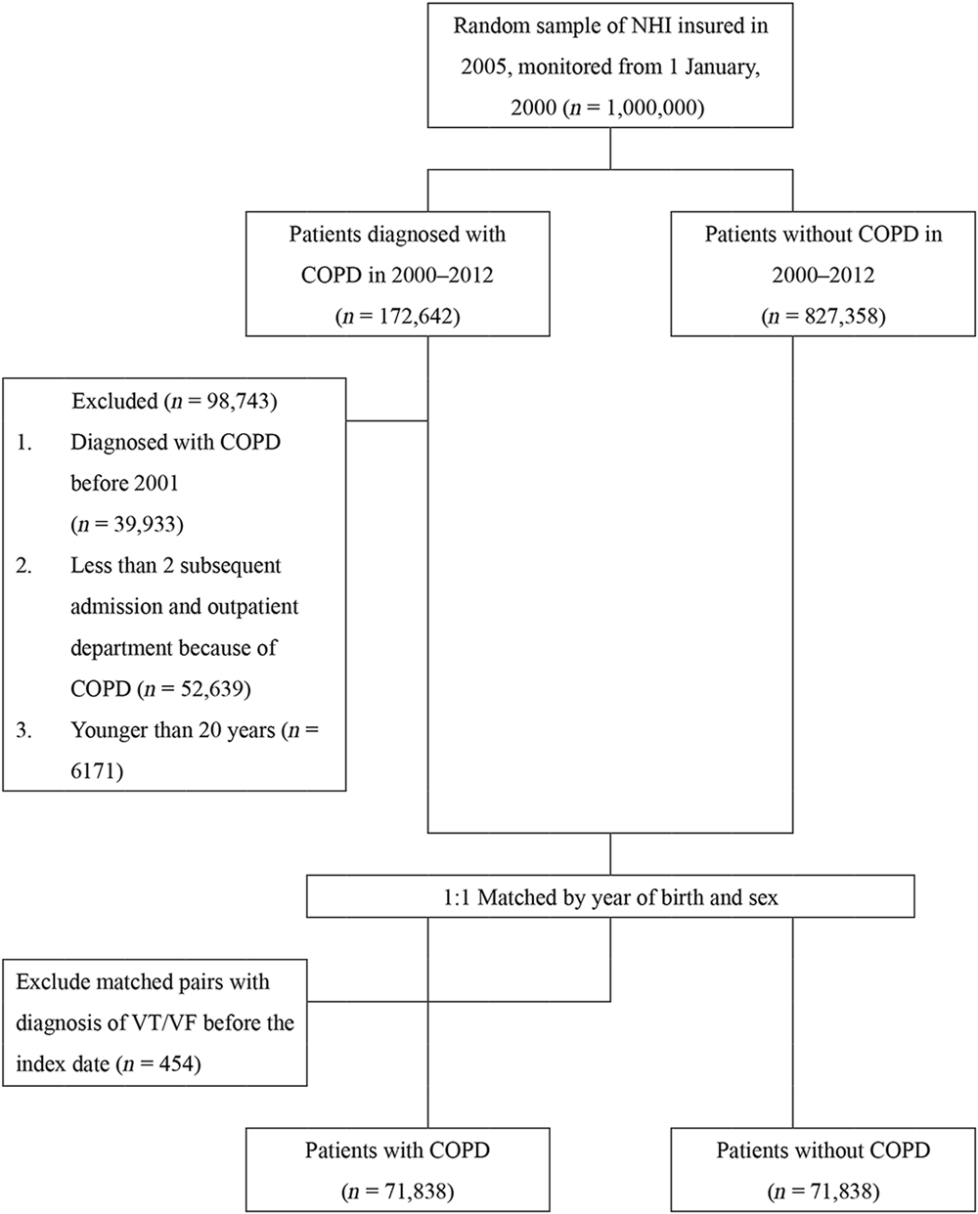
Data selection process. Patients with newly diagnosed COPD since 2001 were matched with controls without COPD at the same index date as the date of diagnosis of COPD.

Records of 172,642 patients with COPD were retrieved, of which 71,838 patients met the inclusion criteria (mean age: 57.66 ± 16.61 years, 54% men). The control cohort was matched with the COPD cohort according to sex and age. The total follow-up duration was 491,198.3 and 497,038.3 person-years in the patient and control cohorts, respectively (Fig. 1).

The baseline characteristics are shown in Table 1. Compared with the control cohort, patients with COPD had a higher prevalence of HF (3.77% vs. 7.44%, *p* < 0.001), AMI (0.96% vs. 1.38%, *p* < 0.001), stroke (10.96% vs. 16.85%, *p* < 0.001), ischemic heart disease (16.21% vs. 24.94%, *p* < 0.001), peripheral vascular disease (6.73% vs. 7.96%, *p* < 0.001), hypertension (34.96% vs. 43.77%, *p* < 0.001), diabetes (17.22% vs. 22.13%, *p* < 0.001), and renal failure (7.58% vs. 10.86%, *p* < 0.001) as well as higher CHA2DS2-VASc scores (score = 1: 27.81% vs. 29.48%; score ≥ 4: 21.70% vs. 24.35%, *p* < 0.001) and ORBIT scores (score = 0–2: 69.68% vs. 74.96%; score ≥ 4: 10.83% vs. 13.19%, *p* < 0.001).

**Table 1:** Characteristic of the sample population.

	Whole cohort (*n* = 143,676)		Non-COPD (*n* = 71,838)		COPD (*n* = 71,838)		P*
	*n*	%	*n*	%	*n*	%	
Age, years (mean ± SD)	57.66 ± 16.61		57.66 ± 16.61		57.67 ± 16.61		0.954
20–64	90,018	62.65	45,022	62.67	44,996	62.64	0.990
65–74	30,354	21.13	15,171	21.12	15,183	21.14	
≥75	23,304	16.22	11,645	16.21	11,659	16.23	
Sex							
Female	65,886	45.86	32,943	45.86	32,943	45.86	0.998
Male	77,790	54.14	38,895	54.14	38,895	54.14	
CHA2DS2-VASc score							
0	30,849	21.47	17,183	23.92	13666	19.02	<0.001
1	42,353	29.48	22,377	31.15	19,976	27.81	
2 or 3	39,298	27.35	18,952	26.38	20,346	28.32	
≥4	31,176	21.70	13,326	18.55	17,850	24.85	
ORBIT score							
0–2	107,706	74.96	57,649	80.25	50,057	69.68	<0.001
3	20,416	14.21	8109	11.29	12,307	17.13	
≥4	15,554	10.83	6080	8.46	9474	13.19	
Comorbidities							
Heart Failure	8049	5.60	2705	3.77	5344	7.44	<0.001
AMI	1684	1.17	691	0.96	993	1.38	<0.001
Stroke	19,976	13.90	7871	10.96	12,105	16.85	<0.001
Ischemic heart disease	29,560	20.57	11,642	16.21	17,918	24.94	<0.001
Angina	10,460	7.28	4061	5.65	6399	8.91	<0.001
Peripheral vascular disease	9665	6.73	3945	5.49	5720	7.96	<0.001
Hypertension	55,984	38.97	24,543	34.16	31,441	43.77	<0.001
Diabetes	28,270	19.68	12,371	17.22	15,899	22.13	<0.001
Depression	4708	3.28	1663	2.31	3045	4.24	<0.001
Renal failure	13,247	9.22	5443	7.58	7804	10.86	<0.001
Chronic liver disease	32,760	22.80	13,273	18.48	19,487	27.13	<0.001
Dementia	4303	2.99	1466	2.04	2837	3.95	<0.001
Medication use							
Aspirin	39,849	27.74	16,244	22.61	23,605	32.86	<0.001
Statin	32,525	22.64	14,394	20.04	18,131	25.24	<0.001
RAASi	52,914	36.83	22,684	31.58	30,230	42.08	<0.001
Class 1 antiarrhythmic drug	2715	1.89	912	1.27	1803	2.51	<0.001
Class 2 antiarrhythmic drug	34,419	23.96	14,948	20.81	19,471	27.10	<0.001
Class 3 antiarrhythmic drug	5277	3.67	1626	2.26	3651	5.08	<0.001
Class 4 antiarrhythmic drug	16,339	11.37	4659	6.49	11,680	16.26	<0.001
Level of urbanization							
Urban	106,310	73.99	53,614	74.63	52,696	73.35	<0.001
Suburban	25,780	17.94	12,720	17.71	13,060	18.18	
Rural	11,586	8.06	5504	7.66	6082	8.47	
Monthly income (US$)							
0	11,052	7.69	5969	8.31	5083	7.08	<0.001
0.03–700	42,830	29.81	20,791	28.94	22,039	30.68	
700–1100	47,714	33.21	23,798	33.13	23,916	33.29	
≥1100	42,080	29.29	21,280	29.62	20,800	28.95	

*AMI* acute myocardial infarction, *COPD* chronic obstructive pulmonary disease, *RAASi* renin–angiotensin–aldosterone system inhibitor.

*The chi-squared test for categorical variables, and *t*-test for continuous variable, two-tailed *p* value.

With regard to the prescribed medication, patients with COPD exhibited a greater use of aspirin, statin, and renin–angiotensin–aldosterone system inhibitors (RAASi) than the controls did. In addition, compared with the control cohort, more patients with COPD were prescribed antiarrhythmic drugs (AADs); this difference was the largest for class II AADs (20.81% vs. 27.10%, *p* < 0.001) and class IV AADs (11.37% vs. 16.26%, *p* < 0.001).

### VA incidence in patients with COPD

**Fig. 2:**
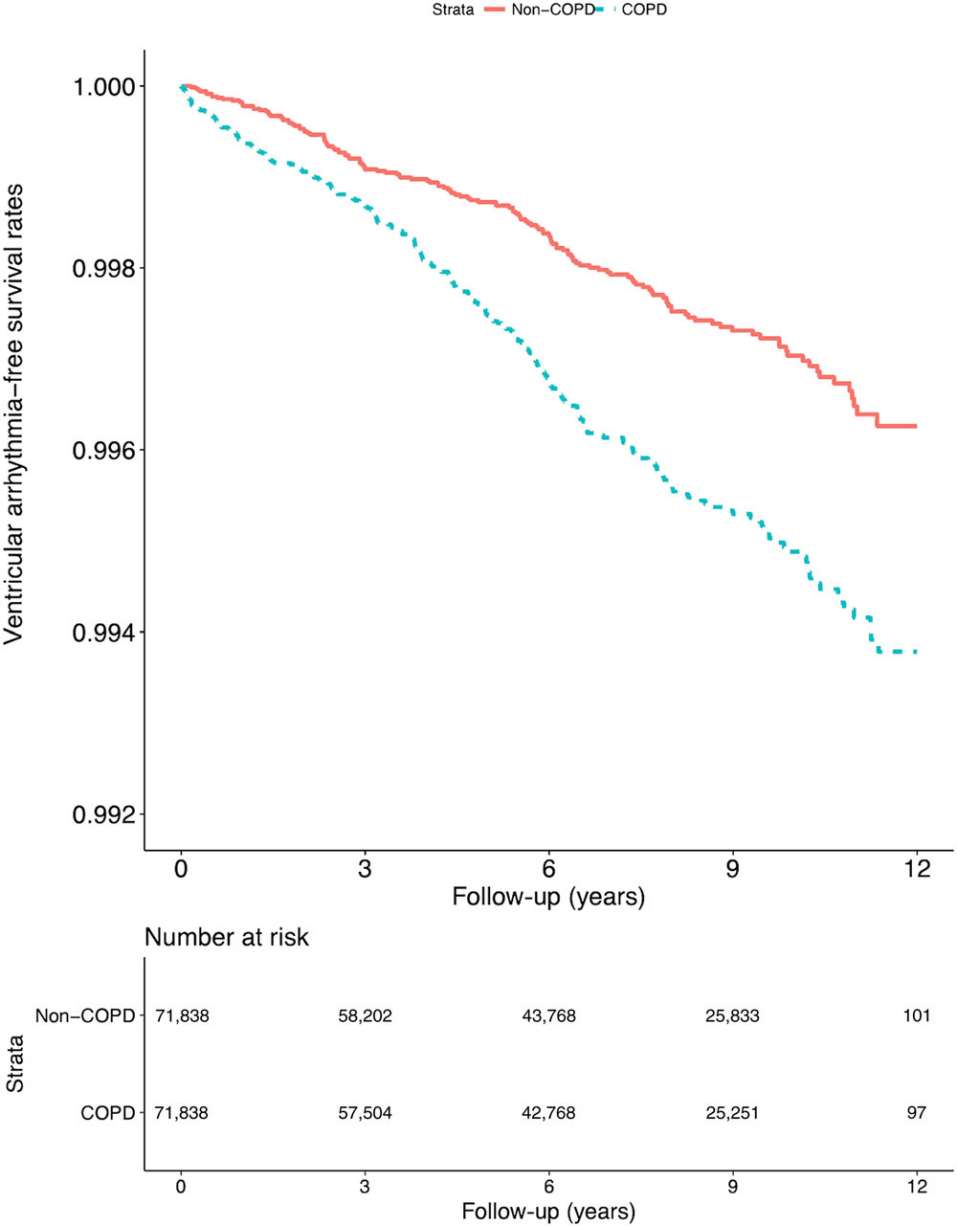
VA admission events in the study cohort (*n* = 143,676) from 1 January 2001 to 31 December 2012 in Taiwan. Data were stratified by COPD and non-COPD (log-rank test, *χ*^2^ = 31.254; df = 1; *p* < 0.001).

The cumulative and relative risks of VA in the patients and controls are presented in Table 2. Compared with the controls, patients with COPD demonstrated higher VA risk during the follow-up period (incidence rates: 57.5 and 98.6 per 10^5^ person-years, respectively; adjusted hazard ratio [aHR]: 1.45; 95% confidence interval [CI]: 1.25–1.68; Fig. 2).

**Table 2:** Risk of VA between the COPD and comparison cohorts.

	Non-COPD (total follow-up 497038.3 person-years)	COPDs (total follow-up 491119.8 person-years)
	Adjusted HR (95% CI)	Adjusted HR (95% CI)
Unadjusted	1.00	1.71 (1.48, 1.98)***
Main model^a^	1.00	1.45 (1.25, 1.68)***
Subgroup effects		
HF		
No	1.00	1.51 (1.28, 1.77)***
Yes	1.00	1.10 (0.75, 1.62)
AMI		
No	1.00	1.45 (1.24, 1.68)***
Yes	1.00	1.47 (0.59, 3.66)
Stroke		
No	1.00	1.42 (1.20, 1.69)***
Yes	1.00	1.47 (1.07, 2.02)*
Ischemic heart disease		
No	1.00	1.65 (1.37, 2.00)***
Yes	1.00	1.12 (0.87, 1.43)***
Angina		
No	1.00	1.48 (1.26, 1.74)***
Yes	1.00	1.19 (0.80, 1.78)
Peripheral vascular disease		
No	1.00	1.47 (1.26, 1.73)***
Yes	1.00	1.23 (0.79, 1.90)
Hypertension		
No	1.00	1.72 (1.35, 2.19)***
Yes	1.00	1.28 (1.06, 1.55)*
Diabetes		
No	1.00	1.48 (1.24, 1.77)***
Yes	1.00	1.36 (1.04, 1.79)*
Renal failure		
No	1.00	1.56 (1.33, 1.84)***
Yes	1.00	0.90 (0.61, 1.31)
CHA2DS2-VASc score		
0	1.00	2.55 (1.48, 4.38)***
1	1.00	1.36 (0.94, 1.95)
2 or 3	1.00	1.46 (1.12, 1.89)**
≥4	1.00	1.26 (1.00, 1.59)*
ORBIT score		
0–2	1.00	1.51 (1.26, 1.82)***
3	1.00	1.24 (0.86, 1.79)
≥4	1.00	1.38 (0.96, 1.97)

*AMI* acute myocardial infarction, *COPD* chronic obstructive pulmonary disease, CI confidence interval.

HF = heart failure; HR = hazard ratio; VA = ventricular arrhythmia.

*: <0.05. **: <0.01. ***: <0.001.

^a^Main model by Cox proportional hazards regression analysis is adjusted for age, sex, CHA2DS2-VASc score, ORBIT score, HF, AMI, stroke, ischemic heart disease, angina, peripheral vascular disease, hypertension, diabetes, depression, renal failure, chronic liver disease, dementia, level of urbanization, and monthly income.

The subgroup effects in the patients with COPD and controls are shown in Table 2. The results showed that after adjustments for the comorbidities, level of urbanization, and monthly income, compared with controls, patients with COPD had higher VA risk (aHR [95% CI]: 1.45 [1.25–1.68]). Compared with the control cohort, patients with COPD without HF, AMI, angina, peripheral vascular disease, or renal failure had a significantly higher VA risk (aHRs [95% CIs]: 1.51 [1.28–1.77], 1.45 [1.24–1.68], 1.48 [1.26–1.74], and 1.47 [1.26–1.73], respectively). All patients with COPD, irrespective of whether they had ischemic heart disease, hypertension and diabetes, or chronic liver disease, had increased VA risk. Patients with COPD with lower CHA_2_DS_2_-VASc scores had higher VA risk (aHRs [95% CIs]: 2.55 [1.48–4.38], 1.36 [0.94–1.95], 1.46 [1.12–1.89], and 1.26 [1.00–1.59] for CHA_2_DS_2_-VASc scores of 0, 1, 2, and 3, ≥4, respectively) than control cohort with same CHA_2_DS_2_-VASc scores. Compared with the control cohort, patients with COPD with lower ORBIT scores also had higher VA risk (aHRs [95% CIs]: 1.51 [1.26–1.82], 1.24 [0.86–1.79], 1.38 [0.96–1.97] for ORBIT scores 0–2, 3, and ≥4, respectively).

### Effect of hospitalization and emergency department visits

Table 3 presents the effects of acute COPD exacerbation on the occurrence of VA. VA risk in patients with COPD increased with each hospitalization or emergency visit because of acute COPD exacerbation. For the first, second, and third visits (whether for hospitalization or an emergency visit), the crude HRs (95% CIs) were 1.35 (1.15–1.59), 3.38 (2.57–4.44), and 4.39 (3.49–5.53), respectively, whereas the aHRs (95% CIs) were 1.29 (1.09–1.52), 1.86 (1.41–2.46), and 2.13 (1.68–2.71), respectively; moreover, after adjustments for the additional drugs prescribed, the aHRs (95% CIs) became 1.28 (1.09–1.52), 1.75 (1.32–2.32), and 1.88 (1.46–2.41), respectively. Notably, the risk remained high in the subgroup analysis when the cohorts were stratified by age or sex. The stratified analysis indicated that in patients with COPD who were hospitalized or made an emergency visit for acute COPD exacerbation, young to middle-aged men had relatively high VA risk. Compared with other patients, young to middle-aged patients with COPD had a higher incidence rate of VA because of acute COPD exacerbation (aHRs [95% CIs] of 20–64-year-old patients: 1.53 [1.17–1.99], 2.04 [1.14–3.65], and 2.91 [1.71–4.94] for the first, second, and third visit, respectively). The men with COPD had a higher incidence rate of VA than did the women with COPD (aHRs [95% CIs]: 1.31 [1.04–1.65], 1.80 [1.25–3.65], and 1.91 [1.45–2.69] for the first, second, and third visit, respectively).

**Table 3:** Risk of lethal VA for frequencies of acute COPD exacerbation.

	Without COPD	Outpatient only	Number of COPD acute exacerbation		
			1	2	≥3
	Adjusted HR (95% CI)	Adjusted HR (95% CI)	Adjusted HR (95% CI)	Adjusted HR (95% CI)	Adjusted HR (95% CI)
Unadjusted^a^	1.00	1.24 (0.82, 1.86)	1.35 (1.15, 1.59)***	3.38 (2.57, 4.44)***	4.39 (3.49, 5.53)***
Main model^a^^,^^b^	1.00	1.18 (0.79, 1.78)	1.29 (1.09, 1.52)**	1.86 (1.41, 2.46)***	2.13 (1.68, 2.71)***
Additional drug^a, c^	1.00	1.19 (0.79, 1.79)	1.28 (1.09, 1.52)**	1.75 (1.32, 2.32)***	1.88 (1.46, 2.41)***
Subgroup effects					
Age, years					
20–64	1.00	1.02 (0.49, 2.10)	1.53 (1.17, 1.99)**	2.04 (1.14, 3.65)*	2.91 (1.71, 4.94)***
65–74	1.00	1.03 (0.50, 2.12)	1.11 (0.83, 1.48)	1.52 (0.96, 2.43)	1.54 (1.01, 2.33)*
≥75	1.00	1.78 (0.89, 3.55)	1.23 (0.88, 1.70)	1.70 (1.08, 2.67)*	1.88 (1.27, 2.77)**
Sex					
Female	1.00	1.20 (0.66, 2.18)	1.25 (0.98, 1.59)	1.69 (1.08, 2.65)*	1.72 (1.12, 2.66)*
Male	1.00	1.17 (0.66, 2.07)	1.31 (1.04, 1.65)*	1.80 (1.25, 2.60)**	1.97 (1.45, 2.69)***
HF					
No	1.00	0.98 (0.60, 1.61)	1.34 (1.12, 1.60)**	1.78 (1.28, 2.49)***	2.24 (1.70, 2.95)***
Yes	1.00	1.82 (0.84, 3.94)	0.93 (0.59, 1.44)	1.42 (0.82, 2.48)	0.87 (0.48, 1.57)
AMI					
No	1.00	1.08 (0.70, 1.67)	1.28 (1.08, 1.52)**	1.84 (1.38, 2.44)***	1.90 (1.47, 2.44)***
Yes	1.00	4.11 (0.93, 18.11)	1.27 (0.46, 3.52)	0.53 (0.06, 4.71)	0.92 (0.17, 4.94)
Stroke					
No	1.00	0.98 (0.59, 1.62)	1.30 (1.07, 1.56)**	1.60 (1.13, 2.28)**	1.78 (1.31, 2.41)***
Yes	1.00	1.83 (0.90, 3.70)	1.17 (0.82, 1.69)	1.96 (1.20, 3.21)**	2.03 (1.30, 3.18)**
Ischemic heart disease					
No	1.00	0.69 (0.34, 1.41)	1.41 (1.14, 1.73)**	2.35 (1.65, 3.36)***	2.51 (1.84, 3.43)***
Yes	1.00	1.64 (0.98, 2.74)	1.03 (0.79, 1.36)	1.11 (0.70, 1.74)	1.11 (0.73, 1.70)
Angina					
No	1.00	0.98 (0.60, 1.60)	1.33 (1.11, 1.58)**	2.02 (1.50, 2.72)***	1.88 (1.42, 2.47)***
Yes	1.00	2.12 (0.97, 4.61)	1.02 (0.65, 1.59)	0.75 (0.31, 1.80)	1.71 (0.94, 3.10)
Peripheral vascular disease					
No	1.00	1.08 (0.69, 1.71)	1.29 (1.08, 1.54)**	1.87 (1.39, 2.52)***	1.89 (1.44, 2.46)***
Yes	1.00	1.85 (0.71, 4.80)	1.19 (0.73, 1.93)	1.02 (0.42, 2.49)	1.75 (0.85, 3.64)
Hypertension					
No	1.00	0.82 (0.36, 1.86)	1.55 (1.19, 2.01)**	2.41 (1.48, 3.91)***	2.18 (1.38, 3.44)***
Yes	1.00	1.35 (0.84, 2.17)	1.11 (0.89, 1.37)	1.44 (1.02, 2.04)*	1.68 (1.25, 2.27)***
Diabetes					
No	1.00	0.72 (0.38, 1.36)	1.32 (1.09, 1.61)**	1.86 (1.32, 2.64)***	2.07 (1.54, 2.78)***
Yes	1.00	2.07 (1.19, 3.60)**	1.18 (0.87, 1.61)	1.55 (0.95, 2.52)	1.45 (0.90, 2.32)
Renal failure					
No	1.00	0.71 (0.40, 1.28)	1.40 (1.17, 1.67)***	2.02 (1.49, 2.73)***	2.17 (1.66, 2.84)***
Yes	1.00	2.83 (1.52, 5.27)**	0.75 (0.49, 1.16)	0.79 (0.37, 1.72)	0.76 (0.36, 1.58)
CHA_2_DS_2_-VASc score					
0 or 1	1.00	0.40 (0.10, 1.61)	1.50 (1.09, 2.07)*	2.51 (1.27, 4.94)**	2.93 (1.62, 5.30)***
2 or 3	1.00	1.30 (0.88, 1.58)	1.18 (0.88, 1.58)	2.17 (1.34, 3.49)**	2.16 (1.41, 3.28)***
≥4	1.00	1.47 (0.84, 2.57)	1.16 (0.90, 1.51)	1.30 (0.87, 1.95)	1.39 (0.97, 2.01)
ORBIT score					
0–2	1.00	0.79 (0.42, 1.49)	1.38 (1.13, 1.69)**	1.93 (1.32, 2.82)***	2.18 (1.57, 3.04)***
3	1.00	1.27 (0.54, 2.99)	1.00 (0.66, 1.51)	1.09 (0.54, 2.20)	1.71 (1.00, 2.92)*
≥4	1.00	2.16 (1.05, 4.46)*	1.13 (0.75, 1.71)	1.75 (1.02, 3.01)*	1.33 (0.77, 2.30)

*AMI* acute myocardial infarction, *COPD* chronic obstructive pulmonary disease, *CI* confidence interval, *HF* heart failure, *HR* hazard ratio, *VA* ventricular arrhythmia.

*: < 0.05. **: <0.01. ***: <0.001.

^a^Cox proportional hazards regression analysis.

^b^Main model is adjusted for age, sex, CHA2DS2-VASc score, ORBIT score, HF, AMI, stroke, ischemic heart disease, angina, peripheral vascular disease, hypertension, diabetes, depression, renal failure, chronic liver disease, dementia, level of urbanization, and monthly income.

^c^Additional drug: use of additional drugs such as Class 1, Class 2, Class 3, and Class 4 antiarrhythmic drugs as well as Aspirin, Stain, and renin–angiotensin–aldosterone system inhibitor. This was included in the model.

Patients who never had to visit the emergency department or get admitted because of acute exacerbation (COPD on an outpatient basis alone) did not have a significantly increased VA risk compared with the controls, except for patients with a history of diabetes and renal failure (aHRs [95% CIs]: 2.07 [1.19–3.60] and 2.83 [1.52–5.27], respectively).

**Fig. 3:**
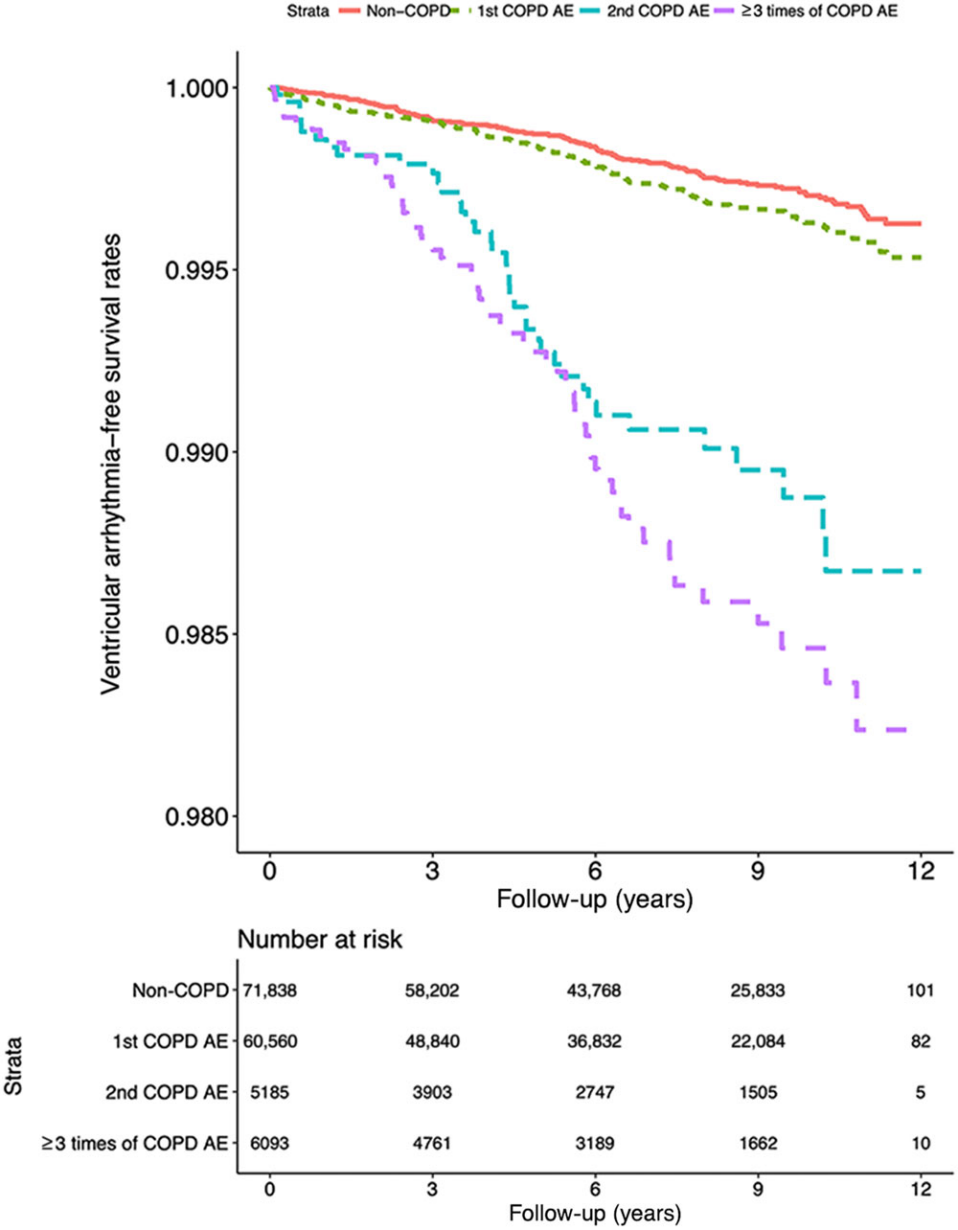
VA admission events in the study cohort (n = 143676) from January 1, 2001 to December 31, 2012 in Taiwan. Data were stratified by the frequency of COPD acute exacerbations (log-rank test, χ^2^ = 198.964; df = 3; p < 0.001).

The frequency of acute exacerbation had more impact on patients without HF, AMI, stroke, ischemic heart disease, angina, peripheral vascular disease, diabetes, and renal failure (aHRs [95% CIs] at the first, second, and third instance of acute exacerbation: 1.34 [1.12–1.60], 1.78 [1.28–2.49], and 2.24 [1.70–2.95], respectively; 1.28 [1.08–1.52], 1.84 [1.38–2.44], and 1.90 [1.47–2.44], respectively; 1.41 [1.14–1.73], 2.35 [1.65–3.36], and 2.51 [1.84–3.43], respectively; 1.33 [1.11–1.58], 2.02 [1.50–2.72], and 1.88 [1.42–2.47], respectively; 1.29 [1.08–1.54], 1.87 [1.39–2.52], 1.89 [1.44–2.46], respectively; 1.32 [1.09–1.61], 1.86 [1.32–2.64], and 2.07 [1.54–2.78], respectively; and 1.40 [1.17–1.67], 2.02 [1.49–2.73], 2.17 [1.66-2.84], respectively). More visits to the emergency department or hospitalizations because of acute COPD exacerbation were associated with lower VA-free survival rate during follow-up (Fig. 3).

### Analysis of patients with ACO

**Fig. 4:**
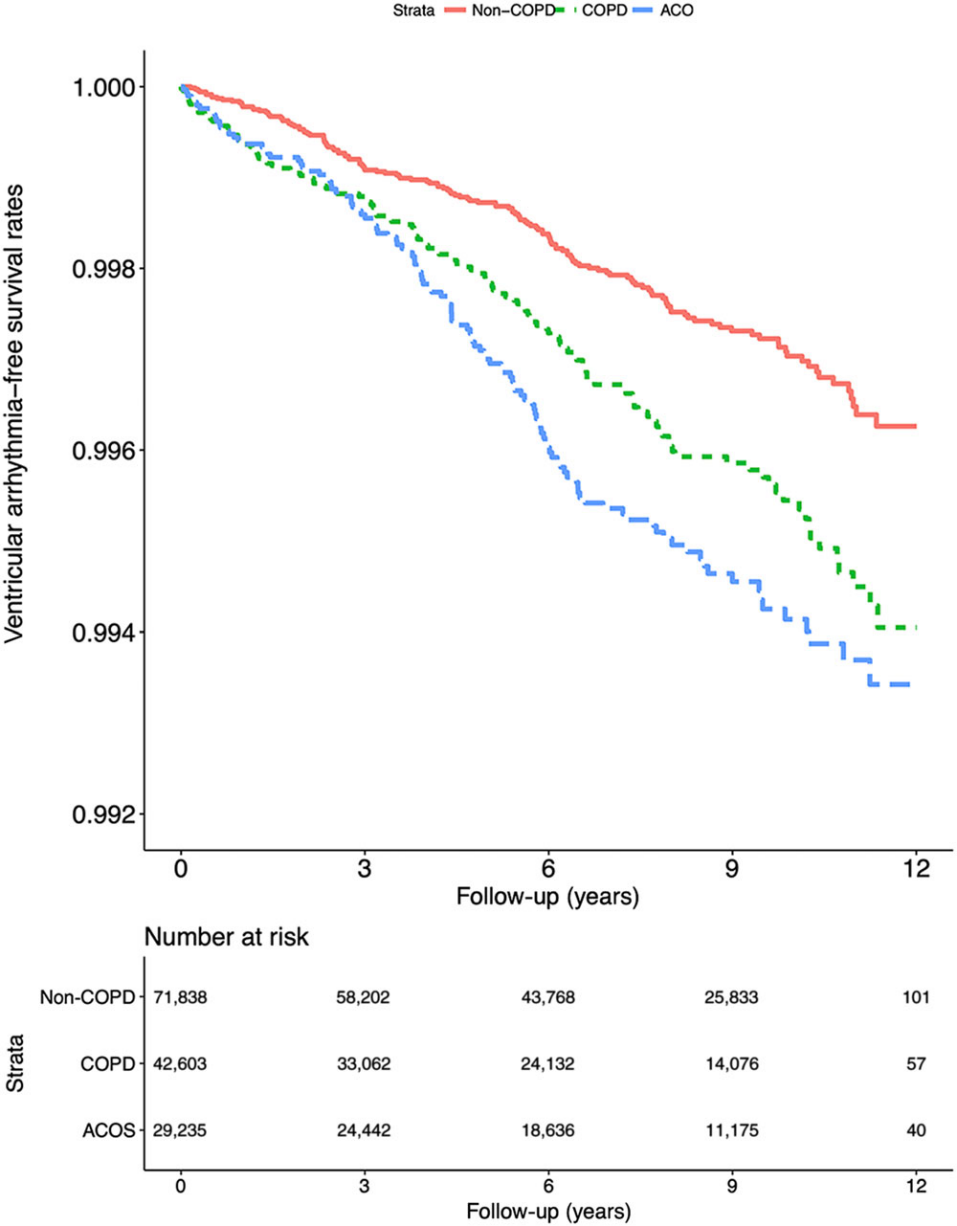
VA admission events in the study cohort (*n* = 143,676) from 1 January 2001 to 31 December 2012 in Taiwan. Data were stratified as non-COPD, COPD alone, and ACO (log-rank test, *χ*^2^ = 34.859; df = 2; *p* < 0.001).

Compared with patients diagnosed as having COPD alone, those diagnosed as having ACO had higher VA risk (aHR [95% CI]: 1.55 [1.30–1.85]; Table 4). The risk remained higher in the ACO group after adjustments for the medication prescribed (aHR [95% CI]: 1.49 [1.25–1.79]). The subgroup analysis revealed that young to middle-aged patients with ACO had higher VA risk than did older patients (aHRs [95% CIs]: 1.67 [1.23–2.27] and 1.45 [1.05–2.01], respectively). Male patients demonstrated higher VA risk than did female patients (aHRs [95% CI]: 1.60 [1.26–2.04] and 1.48 [1.14–1.91], respectively). Patients without HF, AMI, ischemic heart disease, angina, peripheral vascular disease, and renal failure had significantly VA risk (aHRs [95% CIs]: 1.71 [1.41–2.06], 1.56 [1.31–1.87], 1.76 [1.41–2.21], 1.59 [1.31–1.92], 1.56 [1.29–1.88], and 1.68 [1.39–2.04], respectively). VA risk was significantly higher in patients with and without hypertension, diabetes, or stroke. However, higher VA risk was observed in patients without these diseases (aHRs [95% CIs] for those with and without hypertension, diabetes, and stroke: 1.39 [1.35–2.43] and 1.81 [1.35–2.43], respectively; 1.44 [1.05–1.98] and 1.59 [1.29–1.96], respectively; and 1.50 [1.05–2.15] and 1.55 [1.27–1.90], respectively). Patients with ACO exhibited poorer long-term outcomes than those with COPD did (Fig. 4).

**Table 4:** Risk of VA with respect to asthma–COPD overlap.

	Without COPD	Types of airway disease	
		COPD-only	ACO
	Adjusted HR (95% CI)	Adjusted HR (95% CI)	
Unadjusted^a^	1.00	1.48 (1.25, 1.76)***	2.02 (1.70, 2.39)***
Main model^a^^,^^b^	1.00	1.36 (1.14, 1.62)***	1.55 (1.30, 1.85)***
Additional drug^a, c^	1.00	1.32 (1.11, 1.57)**	1.49 (1.25, 1.79)***
Subgroup effects			
Age, years			
20–64	1.00	1.56 (1.18, 2.07)**	1.67 (1.23, 2.27)***
65–74	1.00	1.00 (0.72, 1.37)	1.52 (1.14, 2.03)**
≥75	1.00	1.57 (1.15, 2.14)**	1.45 (1.05, 2.01)*
Sex			
Female	1.00	1.25 (0.96, 1.63)	1.48 (1.14, 1.91)**
Male	1.00	1.45 (1.15, 1.82)**	1.60 (1.26, 2.04)***
HF			
No	1.00	1.35 (1.12, 1.63)**	1.71 (1.41, 2.06)***
Yes	1.00	1.26 (0.82, 1.94)	0.97 (0.62, 1.51)
AMI			
No	1.00	1.35 (1.13, 1.61)***	1.56 (1.31, 1.87)***
Yes	1.00	1.80 (0.66, 4.90)	1.17 (0.40, 3.43)
Stroke			
No	1.00	1.31 (1.07, 1.60)**	1.55 (1.27, 1.90)***
Yes	1.00	1.45 (1.01, 2.08)*	1.50 (1.05, 2.15)*
Ischemic heart disease			
No	1.00	1.57 (1.26, 1.95)***	1.76 (1.41, 2.21)***
Yes	1.00	1.02 (0.77, 1.37)	1.21 (0.92, 1.60)
Angina			
No	1.00	1.40 (1.16, 1.69)***	1.59 (1.31, 1.92)***
Yes	1.00	1.10 (0.68, 1.77)	1.28 (0.81, 2.01)
Peripheral vascular disease			
No	1.00	1.40 (1.17, 1.68)***	1.56 (1.29, 1.88)***
Yes	1.00	1.07 (0.64, 1.81)	1.39 (0.84, 2.29)
Hypertension			
No	1.00	1.66 (1.26, 2.18)***	1.81 (1.35, 2.43)***
Yes	1.00	1.18 (0.94, 1.48)	1.39 (1.11, 1.72)**
Diabetes			
No	1.00	1.39 (1.13, 1.71)**	1.59 (1.29, 1.96)***
Yes	1.00	1.30 (0.94, 1.78)	1.44 (1.05, 1.98)*
Renal failure			
No	1.00	1.47 (1.21, 1.77)***	1.68 (1.39, 2.04)***
Yes	1.00	0.86 (0.55, 1.34)	0.94 (0.60, 1.48)
CHA2DS2-VASc score			
0 or 1	1.00	1.80 (1.30, 2.51)***	1.47 (0.99, 2.18)
2 or 3	1.00	1.08 (0.78, 1.49)	1.88 (1.41, 2.51)***
≥4	1.00	1.28 (0.98, 1.67)	1.25 (0.96, 1.64)
ORBIT score			
0–2	1.00	1.31 (1.05, 1.63)*	1.78 (1.43, 2.21)***
3	1.00	1.19 (0.78, 1.82)	1.30 (0.86, 1.97)
≥4	1.00	1.60 (1.08, 2.37)*	1.14 (0.75, 1.75)

*ACO* asthma–COPD overlap, *AMI* acute myocardial infarction, *COPD* chronic obstructive pulmonary disease, *CI* confidence interval, *HF* heart failure, *HR* hazard ratio, *VA* ventricular arrhythmia.

*: <0.05. **: <0.01. ***: <0.001.

^a^Cox proportional hazards regression analysis.

^b^Main model is adjusted for age, sex, CHA2DS2-VASc score, ORBIT score, HF, AMI, stroke, ischemic heart disease, angina, peripheral vascular disease, hypertension, diabetes, depression, renal failure, chronic liver disease, dementia, level of urbanization, and monthly income.

^c^Additional drug: use of additional drugs such as Class 1, Class 2, Class 3, and Class 4 antiarrhythmic drugs as well as Aspirin, Stain, and renin–angiotensin–aldosterone system inhibitor. This was included in the model.

### Analysis of different classes of antiarrhythmic drugs

In the univariate analysis, all patients with COPD who were prescribed class I–IV AADs exhibited high VA risk (HRs [95% CIs] for class I, II, III, and IV: 3.19 [2.16–4.72], 2.00 [1.64–2.44], 6.34 [4.94–8.14], and 2.53 [2.03–3.15], respectively). The only available class III AAD in Taiwan is amiodarone (Table 5).

**Table 5:** Predictors of new-onset VA in COPD patients.

Exposure variable	Univariate analysis				Multivariate analysis^a^			
	P^*^	HR	95% CI		P*	HR	95% CI	
COPD	<0.001	1.766	1.443	2.162	0.002	1.396	1.131	1.723
Age, years								
20–64		1.000				1.000		
65–74	<0.001	3.702	2.893	4.738	0.003	1.625	1.179	2.239
≥75	<0.001	7.825	6.142	9.970	<0.001	2.489	1.687	3.673
Gender								
Female		1.000				1.000		
Male	<0.001	1.497	1.224	1.830	<0.001	1.692	1.344	2.129
Comorbidities								
HF	<0.001	6.486	5.078	8.286	<0.001	2.375	1.795	3.144
AMI	<0.001	5.529	3.351	9.121	0.022	1.845	1.092	3.117
Stroke	<0.001	3.895	3.144	4.826	0.003	1.489	1.147	1.932
Ischemic heart disease	<0.001	2.919	2.383	3.574	0.371	0.880	0.666	1.164
Angina	<0.001	2.870	2.176	3.785	0.296	1.193	0.856	1.663
Peripheral vascular disease	<0.001	2.661	1.972	3.590	0.388	1.148	0.839	1.572
Hypertension	<0.001	4.406	3.571	5.436	0.001	1.666	1.220	2.275
Diabetes	<0.001	2.981	2.432	3.654	<0.001	1.644	1.294	2.088
Depression	0.946	1.022	0.545	1.915	0.233	0.679	0.360	1.282
Renal failure	<0.001	2.972	2.302	3.837	0.065	1.296	0.984	1.708
Chronic liver disease	0.445	1.098	0.864	1.395	0.082	0.802	0.625	1.029
Dementia	0.002	2.326	1.363	3.972	0.068	0.597	0.344	1.038
Medication use								
Aspirin	<0.001	3.044	2.501	3.705	0.188	1.174	0.924	1.492
Statin	0.020	1.284	1.040	1.586	0.070	0.808	0.642	1.017
RAASi	<0.001	2.903	2.365	3.562	0.680	1.055	0.818	1.360
Class 1	<0.001	3.196	2.163	4.722	0.358	1.213	0.804	1.829
Class 2	<0.001	2.005	1.647	2.441	0.658	0.950	0.758	1.191
Class 3	<0.001	6.349	4.947	8.148	<0.001	2.490	1.885	3.288
Class 4	<0.001	2.530	2.030	3.154	0.737	0.959	0.751	1.225
CHA2DS2-VASc score								
0		1.000				1.000		
1	0.408	1.210	0.770	1.903	0.547	1.161	0.714	1.889
2 or 3	<0.001	3.895	2.618	5.794	0.206	1.431	0.821	2.496
≥4	<0.001	10.133	6.891	14.898	0.523	1.272	0.608	2.660
ORBIT score								
0–2		1.000				1.000		
3	<0.001	1.973	1.528	2.548	0.924	1.013	0.774	1.326
≥4	<0.001	4.121	3.220	5.275	0.414	1.132	0.841	1.524
Level of urbanization								
Urban		1.000				1.000		
Suburban	0.005	1.415	1.114	1.799	0.394	1.114	0.869	1.428
Rural	<0.001	1.750	1.291	2.373	0.091	1.326	0.956	1.839
Monthly income (US$)								
0		1.000				1.000		
0.03–700	0.053	0.758	0.572	1.004	0.087	0.776	0.580	1.037
700–1100	<0.001	0.319	0.234	0.436	<0.001	0.487	0.345	0.686
≥1100	<0.001	0.191	0.133	0.275	<0.001	0.468	0.310	0.706

*AMI* acute myocardial infarction, *COPD* chronic obstructive pulmonary disease, *CI* confidence interval, *HF* heart failure, *HR* hazard ratio, *RAASi* renin–angiotensin–aldosterone system inhibitor, *VA* ventricular arrhythmia.

*Cox proportional hazards regression analysis.

^a^Additional drug: use of additional drugs such as Class 1, Class 2, Class 3, and Class 4 antiarrhythmic drugs as well as Aspirin, Stain, and RAASi. This was included in the model.

### Effect of COPD inhalation medications

No significantly increased VA risk was observed in patients with COPD who had been prescribed short-acting beta-agonists (SABAs), long-acting beta-agonists (LABAs), long-acting muscarinic antagonists (LAMAs), inhalation corticosteroids (ICSs), or LABA–ICS combination therapy. Patients with COPD who were prescribed SABA–short-acting muscarinic antagonist (SAMA) combination therapy had lower VA risk (HR [95% CI]: 0.62 [0.47–0.82]; Supplementary Table).

### Predictor of new-onset VA in patients with COPD

The multivariate analysis indicated that new-onset VA risk was significantly higher in patients with COPD aged 65–74 (HR [95% CI]: 1.62 [1.17–2.23], *p* = 0.003) or ≥75 (HR [95% CI]: 2.48 [1.68–3.67], *p* < 0.001) years; male patients (HR [95% CI]: 1.69 [1.34–2.12], *p* < 0.001); and patients with HF (HR [95% CI]: 2.37 [1.79–3.14], *p* < 0.001), AMI (HR [95% CI]:1.84 [1.09–3.11], *p* = 0.02), stroke (HR [95% CI]: 1.48 [1.14–1.93], *p* = 0.003), hypertension (HR [95% CI]: 1.66 [1.22–2.27], *p* = 0.001), diabetes (HR [95% CI]: 1.64 [1.29–2.08], *p* < 0.001), and history of class III AAD use (HR [95% CI]: 2.49 [1.88–3.28], *p* < 0.001) (Table 5).

## Discussion

The present nationwide population-based study showed that compared with the controls, patients with COPD had a significantly higher VA incidence after adjustments for comorbidities, medications, monthly income, and urbanization level. In addition, VA occurrence in patients with COPD increased with the frequency of hospitalization or emergency department visits. Further, the incidence of VA increases with the complexity of airway diseases. Finally, age, chronic or acute heart disease, stroke, hypertension, and amiodarone prescription were VA predictors in patients with COPD. To the best of our knowledge, the present study involved the largest COPD cohort that has been investigated for VA risk thus far.

Studies have demonstrated high VA risk in patients with COPD^7,9^. COPD is also associated with sudden cardiac death^10^. According to 24-h Holter recordings, patients with COPD had a higher prevalence of sustained or nonsustained ventricular tachycardia, and COPD severity was associated with the burden of ventricular tachycardia^7,9^. In the present study, compared with controls, patients with COPD were at higher risk of developing VA in the future, and the risk was not affected by comorbidities or medication. The autonomic system, hypoxemic status, and beta-agonist inhalation are factors that cause arrhythmogenicity in patients with COPD^11–13^. In addition, a significantly higher T-wave peak-to-end interval also puts patients with COPD into proarrhythmic status^14,15^.

The majority of inhalation medications for COPD used in the present study were not associated with increased fatal VA risk. Bronchodilators could increase supraventricular arrythmia risk, but they did not increase the risk of fatal arrythmias such as ventricular fibrillation, ventricular flutter, or sudden cardiac death^13^. However, we observed that SABA–SAMA combination therapy helped reduce VA risk. This could be attributed to several reasons. First, the combination of SABA–SAMA inhalation medication was administered mainly during the acute exacerbation stage of COPD. Relief of bronchoconstriction and prevention of further respiratory complications might reduce the risk of occurrence of cardiovascular complications, including VA. Second, SABA–SAMA combination therapy could effectively reduce the hyperdynamic inflation in COPD, which might improve cardiovascular outcomes^16–19^.

In the present study, VA risk increased with the frequency of emergency department visits or hospitalization. According to Konecny et al.^9^, nonsustained or sustained ventricular tachycardia risk increased with COPD severity. In addition to the determination of pulmonary function through spirometry, increased episodes of acute COPD exacerbation was associated with poorer outcomes^20^. Hirayama et al.^21^ demonstrated that the risk of hospitalization or emergency events related to atrial fibrillation increased with the frequency of acute COPD exacerbations. In addition, patients with COPD who had no history of emergency department visits or hospitalizations because of acute exacerbation did not show increased VA incidence. Therefore, maintaining a stable status and avoiding episodes of acute exacerbation are essential for reducing VA risk. Furthermore, the present study reported different outcomes in patients with different complexities of airway diseases. Compared with patients with COPD alone, patients with ACO had higher VA risk, and the effects of the complexity remained slightly high after adjustments for comorbidities and medications. A study demonstrated that patients with ACO were more likely to be young; female; and have multiple comorbidities, high obesity risk, or low socioeconomic status^22^. Despite the conflicting results reported in the literature, the long-term mortality, morbidity, and decline of lung function remained critical concerns in patients with ACO^22^. Yeh et al.^23^ demonstrated that compared with patients without ACO, those with ACO had higher cardiac arrhythmia risk, although the types of arrhythmia were not distinguished. To the best of our knowledge, the present study is the first to focus on the effects of ACO on VA occurrence. The results of this study suggest that a more detailed analysis of the relationship between the complexity of lung disease and arrhythmia is required.

CHA_2_DS_2_-VASc scores are known to predict ischemic stroke risk in patients with atrial fibrillation. Moreover, a previous study validated the utility of CHA_2_DS_2_-VASc scores for predicting major adverse cardiovascular event risk in patients with COPD^24^. In the present study, patients with lower CHA_2_DS_2_-VASc scores or ORBIT scores had significant high VA risk than non-COPD patients with same score. While both scores increased, the difference of risk of VA occurrence became less even non-significant. This indicated that in patients with less comorbidities, the association between COPD and unstable VA became more significant. In the further multivariate analysis, CHA_2_DS_2_-VASc scores or ORBIT scores had no significant difference for predicting new-onset VA in patients with COPD.

The multivariate analysis indicated that age >65 years; male sex; previous HF; and a history of stroke, AMI, hypertension, and diabetes mellitus predicted VA risk in patients with COPD. These risk factors are also well-known factors associated with atherosclerotic disease. VA is a complication that occurs in patients with CAD^25^. The major therapies for VA include AAD use, implantable cardioverter defibrillators, and catheter ablation^25,26^. Notably, in our study cohort, the use of class III AADs in patients with COPD was associated with high VA risk. The only class III AAD available in Taiwan is amiodarone, which is widely used for treating arrhythmia in patients with COPD, particularly in patients who cannot tolerate beta-blockers or calcium channel blockers. The association between amiodarone and high VA risk could have several possible explanations. First, amiodarone was reported to exhibit pulmonary toxicity, which may occur even at low doses^27^. A study reported that arrhythmia risk may increase with a decline in respiratory function in patients with COPD, even if their left ventricular function is intact^28^. Second, amiodarone is an AAD that can cause prolongation of the QT interval. In patients with COPD, an increase in the severity of disease is associated with a prolonged QT interval^29^. In addition, the QT interval was noted to be associated with mortality, and it is significantly prolonged in the acute stage of COPD^30^. Excess prolongation of QT intervals increases the risk of VAs such as torsades de pointes^31^.

The present study has several limitations. First, this was a retrospective study, and information on several critical factors, such as direct measurements of pulmonary function and COPD stage, could not be obtained. Rather than direct measurements of COPD severity, emergency department visit and hospitalization frequencies were considered, because acute COPD exacerbation is an indication of COPD severity. In addition, the presence of ACO indicated the complexity of the disease. Second, other possible causes of VA, such as acid–base status, electrolyte levels, renal function, and levels of cardiac biomarkers (e.g., troponin, creatinine kinase, and B-type natriuretic peptide) could not be determined. Using propensity matching, most of the diseases associated with abnormalities in the aforementioned laboratory data were adjusted and equalized in both cohorts. In addition, biomarkers have limited clinical applicability and debatable utility as predictors of sudden death^32^. Finally, some medications that have been demonstrated to reduce VA risk, such as angiotensin–neprilysin inhibitors^33^, were not investigated in the present study, because these drugs were not available in Taiwan from 2008 to 2012. We adjusted for all other medications that had been used for the treatment of cardiovascular disease according to the applicable guidelines^34–37^.

In conclusion, in the present nationwide population-based cohort study, the presence of COPD, acute COPD exacerbation, and airway disease complexity were positively associated with VA risk. In addition to risk factors that are similar to CAD, male and class III AAD use were found to be crucial associated risk factors for VA.

## Methods

The National Health Insurance (NHI) program, established in 1995, provides health insurance coverage to >98% of the population of Taiwan (which approximately 23 million). The NHI Research Database (NHIRD) has been extensively analyzed and validated previously^38,39^. The NHIRD research committee and the Joint Institutional Review Board of Taipei Medical University approved our study protocol (TMU-JIRB No. N201905004) and waived the need for informed consents from participants. This waiver does not affect the rights and welfare of the participants.

### Study cohort

Patients were included in this study if they were diagnosed as having COPD between 1 January 2001 and 31 December 2012, had at least two COPD diagnoses as inpatients or outpatients, and were aged >20 years. A control cohort was selected by blinding the outcome, using SAS (version 9.4, SAS, Cary, NC, USA). Each patient with COPD was matched for birth date and sex with a control who did not have COPD, to compare the incidence of VA. Any individual with prior VA in a matched pair was excluded. In total, 143,676 matched pairs were obtained, comprising individuals in the COPD and control cohorts. The cohort entry date (index date) for patients with COPD was defined as the date of first COPD diagnosis. ACO was defined as COPD diagnosis with a concurrent diagnosis of asthma, with at least two outpatient visits or one admission^40^. The matched pair had the same date of diagnosis of COPD (index date) for follow-up. All patients were followed up until one of the following occurred: an initial diagnosis of ventricular tachycardia (ICD-9-CM code 427.1), ventricular fibrillation, or ventricular flutter (ICD-9-CM code 427.4, 427.41, or 427.42); loss to follow-up; death; withdrawal from the NHI program; or 31 December 2012. Patients who were diagnosed as having only ventricular premature beats (ICD-9-CM code 427.69) during the follow-up period were not enrolled.

### Potential confounders

The cohort was also classified based on sociodemographic characteristics of the participants, such as age (20–64, 65–74, and ≥75 years), sex, urbanization level (urban, suburban, and rural), and monthly income (US$0, US$0.03–US$700, US$700–US$1,100, and ≥US$1,100). The following diagnoses were used to establish the history of baseline comorbidities for each participant: CHA_2_DS_2_-VASc scores—comprising age, sex, congestive HF history, hypertension history, stroke history, vascular disease history, and diabetes history—grouped into four segments (0, 1, 2–3, and ≥4)^41,42^; ORBIT scores—comprising sex, age, bleeding history, glomerular filtration rate (<60 mL/min/1.73 m^2^)— grouped into three segments (0–2, 3, and ≥4)^43^; HF; AMI; stroke; ischemic heart disease; angina; peripheral vascular disease; hypertension; diabetes; depression; renal failure; chronic liver disease; and dementia. Use of the following medications was also controlled for: aspirin; statin; RAASi; and AADs of class I (sodium channel blockers), class II (beta-blockers), class III (potassium channel blockers), and class IV (calcium channel blockers).

We also evaluated the effects of severity of COPD on the occurrence of VA. ACO, a mixed-type airway disease, was evaluated as a risk factor.

### Statistical analysis

The aforementioned baseline patient characteristics are presented in Table 1. Categorical variables were reported as percentages and number of occurrences, and quantitative variables were reported as their mean ± standard deviation. The *t-*test and chi-square test were used to compare the COPD and control cohorts. To determine VA risk in the COPD and control cohorts, a Cox proportional hazards model was used to calculate the HRs and 95% CIs. HRs were adjusted with respect to the aforementioned confounders. Stratified analysis was conducted with the data segmented by age and sex (Table 2). The Cox proportional hazards model was used to estimate the risk of various VA types, with respect to the number of acute exacerbations (Table 3) and complexity (Table 4), in the COPD and control cohorts. Finally, multivariate analysis was used to estimate the association of sociodemographic characteristics, comorbidity, and medications with VA risk (Table 5). Using the Kaplan–Meier method, the VA-free survival rates for the COPD and control cohorts were compared. To examine the effects of hospital admission or emergency department visits (because of acute COPD exacerbation) on VA-free survival rate, we categorized the patients into four groups according to their COPD status (0, 1, 2, and ≥3). To examine the effects of the severity of COPD on VA-free survival rate, we categorized the patients into three groups according to their COPD status: without COPD, with COPD alone, and with ACO. All analyses were performed using SAS, and two-tailed *p* values <0.05 indicated statistical significance.

### Reporting summary

Further information on experimental design is available in the Nature Research Reporting Summary linked to this article.

## Supplementary information

Supplementary Table 1

Reporting Summary

## Data Availability

The data supporting the findings of the present research were sourced from NHIRD in Taiwan. Owing to the legal restrictions imposed by the Government of Taiwan related to the Personal Information Protection Act, the database cannot be made publicly available. However, with reasonable request from authors and with permission from Taiwan NHIRD, the relevant data are available.

## Code availability

No custom codes or software were developed in this study.
