# Supplementary material for: Association between chronic obstructive pulmonary disease and ventricular arrhythmia: a nationwide population-based cohort study
Source: NPJ Prim Care Respir Med. 2021 Feb 12;31:8. doi: 10.1038/s41533-021-00221-3 (PMC7880986; doi:10.1038/s41533-021-00221-3)
Supplement: Supplementary file 1 — Supplementary Table 1 [file 41533_2021_221_MOESM1_ESM.pdf]

Supplementary Table 1

Effect of COPD inhalation medications on new-onset ventricular arrhythmia in COPD patients (n=71838)

Exposure variable	p[a]	HR†	95% C.I.	
SABA	0.253	1.146	0.907	1.447
SABA+SAMA	0.001	0.625	0.474	0.824
LABA (Ultra-LABA)	0.303	0.546	0.173	1.727
LAMA	0.816	0.942	0.571	1.556
ICS	0.637	1.081	0.782	1.494
ICS+LABA	0.138	0.780	0.561	1.083

COPD = chronic obstructive pulmonary disease; ICS: inhalation corticosteroid; LABA: long acting beta-agnonist; LAMA: long acting muscarinic antagonist; SABA: short acting beta-agonist; SAMA: short acting muscarinic antagonist

†Main model is adjusted for COPD medications (SABA, SABA+SAMA, LABA (Ultra-LABA), LAMA, ICS, ICS+LABA), age, sex, CHA2DS2-VASc score, ORBIT score, HF, AMI, stroke, ischemic heart disease, angina, peripheral vascular disease, hypertension, diabetes, depression, renal failure, chronic liver disease, dementia, level of urbanization, and monthly income, Class 1, Class 2, Class 3, and Class 4 antiarrhythmic drugs, Aspirin, Stain, and renin–angiotensin–aldosterone system inhibitor.

[a]Cox proportional hazards regression analysis.
